# Is Concomitant Contralateral Arthroscopic Meniscectomy Effective in Patients Undergoing Unilateral Total Knee Replacement for Knee Osteoarthritis?

**DOI:** 10.3390/jcm15010309

**Published:** 2025-12-31

**Authors:** Kee-Bum Hong, Han-Kook Yoon, Hyun-Cheol Oh, Seungyeon Kang, Sang-Hoon Park

**Affiliations:** Department of Orthopaedic Surgery, National Health Insurance Service Ilsan Hospital, Ilsan-ro 100, Ilsandong-gu, Goyang 10444, Kyunggi-do, Republic of Korea; hongkeebum@nhimc.or.kr (K.-B.H.); hangugy@nhimc.or.kr (H.-K.Y.); hyuncoh@nhimc.or.kr (H.-C.O.); kguin96@naver.com (S.K.)

**Keywords:** total knee arthroplasty, arthroscopic meniscectomy, degenerative osteoarthritis, contralateral knee, clinical outcomes

## Abstract

**Background/Objectives:** In patients requiring unilateral total knee arthroplasty who have relatively mild but symptomatic degenerative osteoarthritis in the contralateral knee, there is ongoing debate regarding whether active intervention, such as arthroscopic surgery, should be performed concurrently or whether conservative management is more appropriate. This study compares patients who underwent simultaneous arthroscopic surgery on the contralateral knee with those who received only conservative treatment, and evaluates the effectiveness of performing arthroscopic surgery concurrently with total knee arthroplasty. **Methods**: From 2007 to 2013, 44 patients underwent unilateral total knee arthroplasty with simultaneous contralateral arthroscopic meniscectomy (Group 1), while 70 patients underwent unilateral total knee arthroplasty and received conservative treatment for degenerative osteoarthritis of the contralateral knee (Group 2). All patients were followed for a minimum of two years. Clinical outcomes were evaluated and compared using the Visual Analog Scale (VAS); Knee Society Score (KSS); and Lysholm score at preoperative, 1-month, 3-month, 1-year, and 2-year postoperative intervals. **Results**: At 1 and 3 months postoperatively, all outcome measures showed improvement compared to preoperative values, with Group 1 demonstrating significantly better results. At 1 and 2 years postoperatively, all three scores remained improved compared to preoperative levels but showed a declining trend relative to the early postoperative period, and no significant differences were observed between the two groups. **Conclusions**: In patients with degenerative osteoarthritis of the knee, simultaneous arthroscopic meniscectomy of the contralateral knee during unilateral total knee arthroplasty was associated with better early outcomes; however, no clinical or statistical differences were observed at 12 months.

## 1. Introduction

Degenerative knee osteoarthritis is a highly prevalent condition, and its management varies depending on symptom severity, functional impairment, and radiographic findings. As degenerative knee osteoarthritis progresses, total knee arthroplasty is generally considered the definitive treatment, and is generally indicated for elderly patients with moderate to severe disease [1,2,3,4,5,6]. However, for patients with mild to moderate osteoarthritis, a variety of treatment options may be considered, including conservative management and arthroscopic procedures [2,3,7,8].

It is common for degenerative knee osteoarthritis to occur bilaterally; however, the severity of arthritis may differ between the two knees, and the rate of progression can vary. Consequently, unilateral total knee arthroplasty is often performed as part of treatment [2,3,4,5,6,8,9,10,11,12]. In patients requiring unilateral total knee arthroplasty, there is ongoing debate regarding the appropriate management of the contralateral knee when degenerative osteoarthritis is present but relatively mild and accompanied by pain. Specifically, it remains controversial whether active interventions such as arthroscopic surgery should be performed concurrently, or if conservative treatment is more appropriate [3,4,5,6,7,8,11,12].

Several biomechanical studies have shown that unilateral total knee arthroplasty can increase mechanical loading on the contralateral knee, which may predispose symptomatic progression in joints with pre-existing degenerative changes. Alnahdi et al. demonstrated that patients exhibit abnormal frontal plane gait mechanics after unilateral total knee arthroplasty, with increased joint loading compared with healthy controls [13]. In addition, Zeni et al. noted that altered gait patterns after total knee arthroplasty are associated with a higher likelihood of requiring contralateral knee arthroplasty [14]. These findings imply that untreated meniscal tears or mild osteoarthritis in the contralateral knee may become clinically significant after unilateral total knee arthroplasty, supporting the rationale for evaluating whether simultaneous arthroscopic intervention offers early symptomatic benefit.

In recent years, multiple randomized controlled trials and meta-analyses have further fueled this debate by suggesting that arthroscopic surgery for degenerative meniscal tears and early osteoarthritis provides minimal long-term benefit compared with conservative treatment, particularly when mechanical symptoms are absent or when cartilage degeneration is advanced. Several studies have reported that, while arthroscopy may offer short-term pain relief or functional improvement, these benefits often diminish by 6 to 12 months, and long-term outcomes do not significantly differ from nonoperative care [15,16,17,18]. In addition, some observational studies have suggested that arthroscopic meniscectomy in older adults may accelerate osteoarthritic progression and potentially lead to an earlier need for arthroplasty [17,18]. These findings have led some guidelines to caution against routine arthroscopy in patients with degenerative knee disease.

In most cases of degenerative osteoarthritis, the prognosis following arthroscopic surgery is reported to depend more on the condition of the articular cartilage than on the meniscectomy itself. Nevertheless, in clinical practice, patients with mild to moderate degenerative osteoarthritis commonly present with meniscal tears accompanied by mechanical symptoms such as catching or locking, which may respond more favorably to arthroscopic intervention. Furthermore, postoperative pain tends to be more severe after total knee arthroplasty compared to arthroscopic surgery, and rehabilitation is generally faster following arthroscopic procedures. Accordingly, in patients with unilateral total knee arthroplasty who also experience significant pain or mechanical symptoms in the contralateral knee, it remains reasonable that simultaneous arthroscopic surgery could improve early postoperative satisfaction and mobility.

Degenerative knee osteoarthritis also represents a substantial public health burden, particularly in aging societies. Epidemiologic studies indicate that more than one-third of adults over the age of 65 experience radiographic or symptomatic knee osteoarthritis [19,20], and many ultimately require surgical intervention when conservative measures fail. In this population, meniscal pathology is highly prevalent and frequently coexists with early degenerative changes, often contributing to pain, mechanical symptoms, and accelerated structural deterioration. MRI-based studies have shown that meniscal tears may precede radiographic joint space narrowing and are strongly associated with the future development of osteoarthritis [21,22], emphasizing the complexity of managing patients with mild radiographic disease but significant soft tissue pathology. These considerations highlight the importance of identifying practical treatment strategies for patients undergoing unilateral total knee arthroplasty who still have symptomatic pathology in the contralateral knee.

With the growing elderly population, determining the most appropriate treatment approach for the contralateral knee in patients undergoing unilateral total knee arthroplasty has become increasingly important. The decision must balance long-term outcomes, recovery profiles, and patient satisfaction, while considering that symptoms in the contralateral knee may worsen postoperatively due to load shifting during early rehabilitation.

In this study, we aimed to evaluate the effectiveness of performing simultaneous arthroscopic surgery on the contralateral knee in patients undergoing unilateral total knee arthroplasty. We compared two groups: one that underwent concurrent arthroscopic surgery on the contralateral knee, and another that received only unilateral total knee arthroplasty with conservative management of the contralateral knee. Our goal was to determine whether simultaneous arthroscopic intervention offers measurable clinical benefits, particularly in terms of early postoperative pain relief and function, in patients with asymmetric bilateral degenerative knee osteoarthritis.

## 2. Patients and Methods

This study included patients who underwent unilateral total knee arthroplasty for degenerative osteoarthritis at Ilsan Hospital between 1 March 2007 and 31 December 2012. Among these, 65 patients who underwent simultaneous arthroscopic surgery on the contralateral knee and were subsequently discharged were included in the analysis. This study was conducted retrospectively, and the study protocol was reviewed and approved by the Institutional Review Board (IRB) of our hospital [NHIMC 2019-07-027, 14 August 2019 approved].

Among the 65 patients initially identified, those who required revision surgery, had incomplete or inaccessible medical records, were followed for less than two years postoperatively, or developed postoperative complications such as infection were excluded. After applying these criteria, 44 patients remained in the simultaneous contralateral arthroscopic meniscectomy group (Group 1).

The control group (Group 2) consisted of 70 patients who underwent unilateral total knee arthroplasty during the same period and received conservative treatment for the contralateral knee. These patients presented with degenerative osteoarthritis of Kellgren–Lawrence grade 1 or 2 and reported knee pain but did not undergo arthroscopic intervention. Conservative management included oral nonsteroidal anti-inflammatory medications, physical therapy, and intra-articular injections when clinically indicated. None of the control group patients underwent operative treatment within the follow-up period.

Primary total knee arthroplasty was performed by three experienced surgeons using a variety of implant systems, including the LPS and LPS-Flex (NexGen; Zimmer Inc., Warsaw, IN, USA), Genesis II (Smith & Nephew, Memphis, TN, USA), and Vanguard (Zimmer Biomet, Warsaw, IN, USA) total knee implants. The indications for total knee arthroplasty included varus malalignment causing functional impairment during ambulation, persistent knee pain unresponsive to conservative management, and radiographic evidence of joint space narrowing corresponding to Kellgren–Lawrence grade 3 or higher on standing weight-bearing radiographs. All surgical procedures were conducted according to standardized operative protocols to ensure optimal implant positioning and soft tissue balance.

The indication for arthroscopic meniscectomy in Group 1 was the presence of degenerative osteoarthritis classified as Kellgren–Lawrence grade 1–2 with a symptomatic meniscal tear confirmed by magnetic resonance imaging (MRI). Arthroscopic treatment was recommended when the patient exhibited mechanical symptoms—such as clicking, grinding, catching, or locking—along with joint line tenderness or a clearly positive McMurray test. The primary source of pain was determined to be mechanical in nature, rather than originating predominantly from joint surface degeneration. All arthroscopic procedures included partial or subtotal meniscectomy of the medial or lateral meniscus, with additional debridement performed as necessary.

Postoperative rehabilitation followed a standardized protocol at our institution. All patients began quadriceps strengthening and ankle pump exercises on postoperative day 1, followed by progressive range of motion exercises as tolerated. Full weight bearing with a walker or crutches was generally allowed within the first weeks unless otherwise contraindicated. Patients in both groups received similar postoperative physiotherapy, allowing the clinical effects of arthroscopic intervention to be evaluated without major differences in rehabilitation intensity.

Clinical assessment was performed through a comprehensive review of electronic medical records. Pain and functional outcomes were assessed using the Visual Analog Scale (VAS), Knee Society Score (KSS), Lysholm score, and range of motion (ROM). These parameters were evaluated preoperatively and at 1 month, 3 months, 1 year, and 2 years after surgery. Major postoperative complications—including infection, deep vein thrombosis, neurovascular injury, and the need for reoperation—were recorded for both groups. Radiographic assessment using weight-bearing knee radiographs—including anteroposterior (AP), lateral, and Rosenberg views—was performed at regular intervals to evaluate progression of osteoarthritis in the contralateral knee.

We also evaluated whether the contralateral knee underwent conversion to total knee arthroplasty during the follow-up period. Incidence of subsequent contralateral knee replacement was tracked for up to five years after the initial surgery, using a combination of electronic medical record review and patient telephone interviews to ensure complete follow-up data.

Statistical analyses were performed using SPSS version 13.0 (SPSS Inc., Chicago, IL, USA). Continuous variables are expressed as the mean and standard deviation. Categorical variables are presented as frequencies. Statistical differences were assessed using paired *t*-tests, chi-square tests, and Mann–Whitney tests. For continuous variables that did not meet parametric assumptions, the nonparametric Wilcoxon rank-sum test was applied. All statistical analyses were conducted at a significance level of 0.05.

## 3. Results

### 3.1. Demographic Data

All patients were followed up for a minimum of 2 years. Group 1 consisted of 44 patients who underwent arthroscopic meniscectomy, while Group 2 included 70 patients who received conservative treatment for the contralateral knee. The baseline demographic characteristics were comparable between the two groups. The mean ages of the two groups were 68.1 and 67.7 years, respectively, with no statistically significant difference, and there was no difference in sex distribution. Body mass index (BMI) also did not differ significantly between the two groups (Table 1). Preoperative Visual Analog Scale (VAS) scores, Knee Society Scores (KSSs), and Lysholm scores all showed no significant differences between the two groups (Table 2, Table 3 and Table 4).

### 3.2. Postoperative Results

Both groups demonstrated a significant reduction in VAS scores at 1 month and 3 months postoperatively when compared with preoperative values. However, Group 1 showed a more pronounced early improvement. At 1 month, Group 1 exhibited a significantly lower VAS score compared to Group 2 (3.5 vs. 6.8, *p* = 0.04). A similar pattern was observed at 3 months (2.1 vs. 4.1, *p* = 0.04). At longer follow-up points (1 year and 2 years), VAS scores increased in both groups relative to the early postoperative period. This deterioration appeared to be associated with symptom progression in the contralateral knee that did not undergo arthroplasty. At 1-year and 2-year follow-ups, no significant differences were found between the two groups (Table 2).

Knee Society Scores (KSS) also improved significantly at 1 and 3 months postoperatively in both groups. Group 1 demonstrated superior functional improvement during the early postoperative period, showing significantly higher scores at both 1 month (85.1 ± 7.2 vs. 75.1 ± 8.0, *p* = 0.02) and 3 months (89.1 ± 8.1 vs. 82.1 ± 7.5, *p* = 0.01). After 1 and 2 years of follow-up, KSS values in both groups decreased compared with the early postoperative period but remained improved relative to preoperative levels. Similarly to the VAS results, no statistically significant differences were observed between the two groups at the 1- and 2-year evaluations (Table 3).

Lysholm scores showed a postoperative pattern similar to that of VAS and KSS. Both groups experienced substantial improvement at 1 and 3 months, with Group 1 again demonstrating superior early outcomes (82.2 ± 7.1 vs. 71.1 ± 6.6 at 1 month, *p* = 0.03; 85.1 ± 6.8 vs. 75.1 ± 6.3 at 3 months, *p* = 0.02). At 1-year and 2-year follow-ups, Lysholm scores declined compared to 1- and 3-month assessments in both groups but remained improved relative to preoperative values. No significant differences were observed between the groups during long-term follow-up (Table 4).

Conversion to contralateral total knee arthroplasty during the follow-up period was evaluated for both groups. Within 2 years after the initial surgery, 12 of 70 patients (17.1%) in Group 2 underwent total knee arthroplasty on the contralateral knee. In Group 1, 7 of 44 patients (15.9%) required contralateral total knee arthroplasty during the same period, and there was no significant difference between the two groups. By the 5-year follow-up, the cumulative conversion rates increased to 32 of 70 patients (45.7%) in Group 2 and 20 of 44 patients (45.5%) in Group 1. No statistically significant differences were observed between the two groups at any of the evaluated time points (Table 5).

No major complications—including infection, neurovascular injury, or thromboembolic events—were observed in either group throughout the entire follow-up period. At the 2-year postoperative follow-up, the mean knee range of motion was 126.3 ± 9.3° in Group 1 and 125 ± 8.3° in Group 2, with no statistically significant difference between the groups.

## 4. Discussion

In this study, performing arthroscopic meniscectomy on the contralateral knee simultaneously with total knee arthroplasty in patients with degenerative osteoarthritis resulted in better early clinical outcomes, including pain relief and functional recovery within the first 3 months postoperatively. However, at the 1- and 2-year follow-ups, symptoms tended to worsen again, and no significant differences were observed between the surgical and conservative treatment groups. There was no statistical difference between the Group 1 and Group 2 patients who underwent total knee arthroplasty on the opposite leg after surgery. It was found that there was no significant difference in patients undergoing total knee replacement surgery between conservative treatment on the other leg or meniscectomy. There was a clear reduction in pain in the early stages. However, it was found that it did not worsen the knee or improve symptoms in long-term follow-up. These findings suggest that, while simultaneous arthroscopic meniscectomy may offer meaningful short-term benefits for selected patients, its long-term impact remains limited, highlighting the need for careful patient selection and further research to determine the clinical situations in which this approach may be most appropriate.

When performing unilateral total knee arthroplasty in patients with degenerative osteoarthritis and meniscal tears in the contralateral knee, there is currently no definitive data to indicate whether arthroscopic meniscectomy or conservative management is superior. In this study, we analyzed clinical outcomes based on the degrees of osteoarthritis in patients who could be followed for more than two years, comparing a group that received conservative treatment with a group that underwent surgical intervention (arthroscopic partial or subtotal meniscectomy). In terms of pain, function, and overall clinical outcomes, the surgical group showed significantly better improvement at 1 and 3 months postoperatively compared to the conservative treatment group. However, at 1 and 2 years of follow-up, both groups demonstrated a gradual decline in outcomes, and no significant differences were observed between them. Although surgical treatment was beneficial for early pain relief and functional recovery, the long-term outcomes were comparable to those of the conservative treatment group. The discrepancy between early postoperative improvement and later convergence of outcomes may be partially explained by the biomechanical effects of partial meniscectomy. Removal of unstable meniscal fragments can temporarily reduce mechanical irritation, thereby improving pain and function in the short term. However, long-term outcomes may depend more heavily on the baseline degenerative status of the cartilage and the continued progression of osteoarthritis, which may offset diminishing early symptomatic gains over time. Nevertheless, after a minimum follow-up of 2 years, both groups showed improvement compared to their preoperative status.

In a recent randomized controlled trial evaluating the efficacy of arthroscopic partial meniscectomy, Yim et al. reported that arthroscopic meniscectomy was not superior to conservative management such as strengthening exercises [4]. Their findings were consistent with the present study, showing no significant difference between the surgical and conservative treatment groups at the 2-year follow-up. Although both groups demonstrated improvement compared to preoperative status, the surgical group exhibited a tendency for worsening VAS scores at 1 and 2 years postoperatively compared to the early postoperative period. In contrast, the conservatively treated group showed gradual improvement. Notably, despite including only patients with degenerative horizontal meniscal tears and no cartilage damage, Yim et al.’s study demonstrated results in line with ours. Similarly, Sihvonen et al. reported that arthroscopic partial meniscectomy showed no significant difference compared with sham surgery [5]. In their study, clinical outcomes at 1 year postoperatively were improved compared to preoperative values; however, VAS and Lysholm scores worsened compared to the early postoperative period (1 and 6 months). In contrast, in our study, surgical treatment demonstrated greater improvement in pain relief and functional recovery during the early postoperative period (1 and 3 months) compared to conservative treatment. These findings suggest that, when surgical indications are appropriately selected, arthroscopic meniscectomy may be beneficial for early pain relief in patients with significant symptoms.

Maxwell et al. reported that preoperative pain in the contralateral knee can negatively affect patient satisfaction after TKA, and that patients with contralateral knee pain may have worse outcomes compared to those undergoing bilateral TKA [6]. From this perspective, active evaluation and management of contralateral knee pain are important to achieve better postoperative outcomes in unilateral TKA. As weight bearing often shifts toward the contralateral knee following surgery, increased mechanical stress during the early recovery period may exacerbate symptoms in the non-operated knee. In the present study, patients who underwent simultaneous contralateral arthroscopic meniscectomy showed superior early postoperative results compared to those who received conservative treatment. Therefore, in cases where contralateral knee osteoarthritis is accompanied by meniscal tears and clear mechanical symptoms, surgical intervention may contribute to higher early postoperative satisfaction and improved short-term outcomes.

Several previous studies have emphasized that radiographic severity of the contralateral knee is a strong predictor of later conversion to TKA following unilateral arthroplasty. Ritter et al. reported that the likelihood of contralateral TKA increased from 21% at 7 years to 37% at 10 years when osteoarthritis changes were present at the same time of index surgery [7]. Similarly, Mont et al. found that 93% of patients demonstrating moderate-to-severe symptoms with advanced radiographic degeneration eventually required contralateral TKA [8]. McMahon and Block further highlighted baseline Kellgren–Lawrence grade as a key determinant of contralateral knee survival, noting that no patients with grade 0–1 progressed to arthroplasty, whereas those with grade 4 had significantly shorter survival times [9]. However, these radiographic grading systems have well-recognized limitations, as they may fail to detect early cartilage loss and may not adequately reflect meniscal pathology [2,3]. Considering that meniscal tears and chondral lesions significantly contribute to symptom progression independent of radiographic grade [21], the inclusion of patients with MRI-confirmed meniscal pathology in our study may account for the earlier symptom worsening and faster conversion to TKA despite relatively mild radiographic changes. These findings highlight the need to evaluate soft-tissue pathology, rather than relying solely on radiographs, when estimating prognosis and determining the optimal management of the contralateral knee in patients undergoing unilateral TKA.

According to the current standards of the National Health Insurance Service in South Korea, total knee arthroplasty is indicated for patients aged 65 years or older with degenerative osteoarthritis of Kellgren–Lawrence grade 3 or 4, whereas patients with grade 1 or 2 osteoarthritis are not eligible for the procedure. When there is a difference in the severity of degenerative osteoarthritis between both knees, total knee arthroplasty may not be feasible on the contralateral side. In such cases, various treatment options may be considered, including conservative management and surgical interventions such as arthroscopic meniscectomy. Total knee arthroplasty is inevitably carried out after holding out until the last stage for as long as possible. It is more desirable to try several other methods first if pain can be relieved and functional improvement can be promoted through various treatment methods. However, clear evidence supporting the optimal treatment approach remains scarce [2,3,4,5,6,8,10,11,12]. In patients with severe pain and functional decline, surgical treatment showed favorable early outcomes. However, even when arthroscopic meniscectomy was performed, degenerative changes accompanied the contralateral knee over time, leading to symptoms worsening at 1 and 2 years postoperatively. From a long-term perspective, conservative management such as pharmacologic treatment may prove to be more beneficial than surgical intervention in terms of cost-effectiveness.

In general, arthroscopic meniscectomy has been reported to yield better outcomes than conservative treatment for patients with meniscal tears, particularly for those presenting with mechanical symptoms [2,3,7,8,9,10,12]. Some studies have also reported superior outcomes with surgical treatment in patients with meniscal tears accompanied by degenerative osteoarthritis [2,4,5,6,7,11,12]. However, recent studies have reported that clinical outcomes are generally poor when meniscectomy is performed in elderly patients [1,2,3,5,7,10]. As the population continues to age and the number of elderly individuals participating in sports increases, there is a growing need for more proactive treatment strategies [2,3,4,5,6,7]. However, compared to younger patients, clinical outcomes of all treatments—including conservative and surgical interventions—tend to be poorer in elderly patients, highlighting the need for further research in this population.

Based on the findings of this study, arthroscopic meniscectomy may be considered concurrently in cases with severe initial pain and functional impairment. However, patients should be informed that at 1- and 2-year follow-ups, the difference between surgical and conservative treatments disappears, and the progression of degenerative osteoarthritis continues. The limitations of this study are as follows. First, it was conducted retrospectively, which carries a risk of selection bias. Additionally, the patient population was relatively elderly, resulting in a small sample size and limited ability for long-term follow-up. Further studies with prospective designs, larger cohorts, and longer follow-up periods are needed to clarify which patient subgroups may benefit most from simultaneous arthroscopic intervention. In particular, incorporating advanced imaging, biomechanical analysis, and patient-reported outcomes may help refine indications and optimize personalized treatment strategies. Additionally, evaluating cost-effectiveness and rehabilitation outcomes could further guide clinical decision-making and support the development of practical applications in routine practice.

## 5. Conclusions

In this study, performing arthroscopic meniscectomy on the contralateral knee simultaneously with unilateral total knee arthroplasty in patients with degenerative knee osteoarthritis resulted in better early clinical outcomes, including pain relief and functional recovery within the first 3 months postoperatively. However, at the 1- and 2-year follow-ups, symptoms tended to worsen again, and no significant differences were observed between the surgical and conservative treatment groups. These findings suggest that, while simultaneous arthroscopic meniscectomy may offer meaningful short-term benefits for selected patients, its long-term impact remains limited, highlighting the need for careful patient selection and further research to determine the clinical situations in which this approach may be most appropriate.

## Figures and Tables

**Table 1:** Demographic factors.

Parameters	Group 1 Meniscectomy (n = 44)	Group 2 Conservative Management (n = 70)	*p*-Value
Age (years)	68.1 ± 4.2	67.7 ± 3.9	0.48
Gender: female/male	41/3	63/7	0.5
Body mass index(kg/cm^2^)	26.4 ± 3.2	26.1 ± 3.6	0.71

Data are presented as means with standard deviation (*p*-value < 0.05).

**Table 2:** Postoperative changes in the VAS score.

Postoperative Day	Group 1 Meniscectomy (n = 44)	Group 2 Conservative Management (n = 70)	*p*-Value
Preop.	9.4 (7–10)	9.3 (4–10)	0.28
1 mos	3.5 (0–8)	6.8 (1–9)	0.04
3 mos	2.1 (0–6)	4.1 (0–5)	0.04
1 yr	4.2 (0–7)	4.4 (0–8)	0.81
2 yrs	6.1 (0–10)	6.3 (0–10)	0.34

Visual Analog Scale (VAS).

**Table 3:** Postoperative changes in the KSS.

Postoperative Day	Group 1 Meniscectomy (n = 44)	Group 2 Conservative Management (n = 70)	*p*-Value
Preop.	67.1 ± 6.9	66.1 ± 7.1	0.21
1 mos	85.1 ± 7.2	75.1 ± 8.0	0.02
3 mos	89.1 ± 8.1	82.1 ± 7.5	0.01
1 yr	76.1 ± 7.2	76.1 ± 5.8	0.34
2 yrs	72.1 ± 8.1	73.1 ± 7.5	0.41

**Table 4:** Postoperative changes in the Lysholm score.

Postoperative Day	Group 1 Meniscectomy (n = 44)	Group 2 Conservative Management (n = 70)	*p*-Value
Preop.	35.8 ± 10.1	35.2 ± 11.6	0.41
1 mos	82.2 ± 7.1	71.1 ± 6.6	0.03
3 mos	85.1 ± 6.8	75.1 ± 6.3	0.02
1 yr	81.1 ± 8.3	72.1 ± 9.3	0.72
2 yrs	76.1 ± 9.8	65.1 ± 10.1	0.81

**Table 5:** Contralateral total knee arthroplasty cases after surgery.

	Group 1 (n = 44)	Group 2 (n = 70)	
Postop 1 yr	3	3	0.87
Postop 2 yrs	4	9	0.75
Postop 3 yrs	3	12	0.19
Postop 4 yrs	3	9	0.48
Postop 5 yrs	7	11	1
Total	20	32	0.08

## Data Availability

The datasets used and analyzed during the current study are available from the corresponding author upon reasonable request.
